# Population structure and phenological attributes of *Adansonia digitata* L. (baobab) in Northwestern lowland area of Ethiopia

**DOI:** 10.1016/j.heliyon.2023.e22571

**Published:** 2023-11-24

**Authors:** Melkamu Abere, Zewdu Yilma, Tadesse Tsegie, Abeje Eshete, Asmamaw Alemu

**Affiliations:** a Ethiopian Forestry Development Bahir Dar Center, P.O. Box 2128, Bahir Dar, Ethiopia; b Ethiopian Forestry Development Central Ethiopia Center, P.O. Box 33042, Addis Ababa, Ethiopia; c Ethiopian Forestry Development, P.O. Box 24536, Addis Ababa, Ethiopia; d University of Gondar, P.O. Box 196, Gondar, Ethiopia

**Keywords:** Baobab, Density, Distribution, Phenological characteristic, Fruit yield

## Abstract

*Adansonia digitata* (baobab), a multipurpose and highly valued tree species, is facing threats due to anthropogenic factors like shifting cultivation practices and fire. The aim of this study was to examine the population structure and phenological attributes of baobab in three districts (i.e. Kafta Humera, Tselemt, and Quara district) in Northwestern Ethiopia. The study was carried out by establishing 17 plots 1 km long and 100 m wide covering a total area of 170 ha in the Quara district and five plots covering 50 ha each in the Kafta Humera and Tselemt districts. Further, plots were subdivided into 25 × 25 m and 5 × 5 m sub-plots for recording other woody species and their regeneration status, respectively. Thirty reproductively matured trees with easily visible crowns were selected to record phenological characteristics and fruit yield. The findings revealed that baobab population was significantly higher in the Tselemt district (3.15 ± 0.15) as compared to Quara (1.43 ± 0.43) and Kafta Humera (1.30 ± 0.23) sites. A bell-shaped diameter distribution was observed in the Quara district and irregular-shaped distributions were observed in Kafta Humera and Tselemt districts. Phenological periodicity and fruit production of baobab did not vary significantly among the three study sites. On average, 404 fruits per tree were recorded with a maximum of 559 fruits in mid-diameter size class trees. Due to livestock browsing, shifting cultivation practices, and uncontrolled fire, the recruitments are limited in the study areas. An in-situ conservation strategy through the plantation and proper management practices are needed to sustain baobab tree species.

## 1 Introduction

*Adansonia digitata* L. (baobab) is a multipurpose wild edible plant, which has traditionally been valued as a source of food, water, health remedies, or places of shelter and supports many animals as a source of key foods. It is a very long-lived tree and has an average life span of 1000–3000 years with a mean of ca. 2000 years [[1], [2], [3], [4]]. Baobab is characterized by deciduous leaves and massive size, reaching a height of 25 m [2,5,6]. The species are distributed widely scattered in the semi-arid and arid regions to the south of the Sahara, throughout Tropical and Sub-Saharan Africa [2,7,8]. It grows abundantly in areas with low rainfall, and high temperature and it is adapted to drought and fire [[9], [10], [11]].

Typically, baobab has functioned as a keystone species making an important contribution to ecosystem functioning and people's livelihoods for food, fiber, and medicine, [[12], [13], [14], [15]]. All different parts of baobab (barks, flowers, fruits, leaves, roots, seed) are traded and used for food, in the traditional pharmacopoeia, traditional rites, and crafts [[16], [17], [18], [19], [20], [21], [22]]. The baobab tree is considered a source of income and employment for rural as well as urban populations [19]. The species has more than 300 uses and commercial values that have been identified in the European Union and the United States [13,23]. Its various products are bartered and sold in urban and informal markets across Africa [15]. Statutory bodies of different countries have approved fruit pulp as a food supplement because of certain nutritional products [24]. In addition of the socioeconomic importance, baobab have ecological and environmental significances in many African countries [16,25].

However, the population of baobab has been declining due to different anthropogenic factors like herbivores, fire, unsustainable land conversion, and removal of seedlings on farmlands [6,22,26]. Besides, baobab seeds are thick, and their dormancy is difficult to break [6,20]. Emerging seedlings also do not effectively survive because of external factors associated with the microsite environment [27]. Lack of consistent rainfall influenced regeneration. This situation is exacerbated by livestock browsing and trampling, resulting in extremely high rates of seedling and sapling mortality [28]. These anthropogenic and ecological factors associated with climatic factors are leading to the loss of the baobab population in the Sahel zones [29].

Despite their ecological, environmental, and economic benefits in Ethiopia, limited research has been done to examine the population structure and reproductive attributes of baobab tree species in some parts of the country [30,31]. This type of study is important to manage sustainably and domestication of the tree species in the study areas. Therefore, the aim of this study was to examine the population structure and phenological attributes of baobab in three study districts (i.e. Kafta Humera, Tselemt, and Quara district) of Northwestern Ethiopia.

## 2 Materials and methods

### 2.1 Description of the study area

**Fig. 1:**
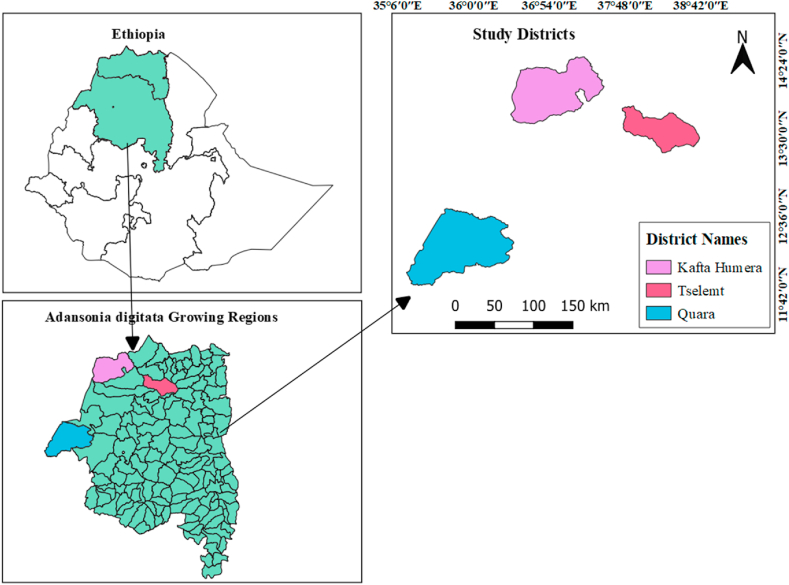
Map showing the baobab study area.

The study was conducted in the Quara, Kafta Humera, and Tselemt districts of the Northwestern lowland area of Ethiopia (Fig. 1). The three study districts are found relatively in a similar agro-ecological zone with similar altitude, temperature, and rainfall. The town of Quara district is found at about 1049 km from Addis Ababa and Kafta Humera and Tselemt district towns are located at about 1200 km and 870 km in North West direction from Addis Ababa town, respectively.

Vegetation of the study sites are known as the *Combretum-Terminalia* woodland vegetation [[32], [33], [34]]. Such woodlands are often found in shallow soils and sandy river valleys [33,35]. The rainfall distribution of all study sites ranges from 400 to 1200 mm. The mean annual temperature of Quara district ranges from 25 to 42 °C and that of Kafta Humera and Tselemt districts ranges from 17.5-41.7 °C. The rainy season of the study areas is from June to September. The remaining 8–9 months between October and May are dry and hot. The maximum temperature is recorded in the three districts in April and May, and the minimum temperature is in August and July. Parent materials of the area are dominated by volcanic felsic and metamorphic Pre-Cambrian basements, and in some parts, Limestone. Lepto soils and Verti soils are the pre-dominant prevailing soil types in the area.

### 2.2 Research design and data collection

Quick reconnaissance surveys were conducted in each study district to identify where baobab tree species grow and to select representative specific sites that harbor the species. In Quara district at Gelegu kebele, Kafta Humera and Tselemt district at Senasil and Dima kebele were selected respectively and purposively. Each selected growing area of baobab was delineated and prepared grid maps on the desktop study using Google Maps. Ten percent of the delineated area was randomly employed as a sampled plot, which was later verified on the ground to be true.

The sampling was designed according to Venter and Witkowski [15] with slight modifications where a plot of 1 km long and 100 m wide (equivalent to 10 ha) was used because baobabs were sparsely distributed. In the field, each main plot was established using a Global Positioning System (GPS). A total of 27 main sample plots, accounting for 270 ha (i.e., 170 ha in the Quara district and 50 ha in each Kafta Humera and Tselemt district) were sampled. To identify and record associated tree species of baobab and their composition, 25 × 25 m subplots were laid systematically in each main sampled plot. For all counted all matured baobab trees, diameter at breast height were measured in each plot. Regeneration of baobab where found in each plot was recorded. Thirty reproductively matured trees with easily visible crowns were selected and marked to study phenological characteristics and fruit production. Continuous observation and data recording on their phenology and fruits were recorded at intervals of 15 days for two consecutive years. The number of fruits per tree and total fruit mass/weight per tree were measured in selected matured trees.

### 2.3 Data analysis

Data were analyzed using SPSS Statistics version 20 and Microsoft 2010. A one-way ANOVA test was used to assess any significant differences in baobab tree density and fruit yield in the different study districts and diameter and height size classes.

Density in different study areas was calculated by summing up all the stems across all sampled plots (abundance) and translating to hectare base for the species encounter in the study plots. Frequency distribution was determined from the number of plots, which a species was recorded (absolute frequency) [36] and as a percentage (relative frequency) by dividing the absolute frequency of the species by the sum of the absolute frequencies. The population structure of baobab tree species was assessed from the frequency distribution of diameters based on a bar graph constructed by grouping into the following successive diameter size classes of ≤50, 51–100, 101–150, 151–200, 201–250, 251–300, 301–350 and 351–400. Size class distributions and phenophase calendar were summarized using graphical and tabular methods respectively.

## 3 Results and discussion

### 3.1 Density of baobab stands

Descriptive statistics of the stand density of baobab in the three study areas are presented in Table 1. The result showed that the stand density of baobab trees significantly differed between study districts (P = 0.002). The highest mean density 3.15 trees per hectare were recorded in the Tselemt district. The second-highest density 1.43 trees per hectare and the lowest density 1.30 trees per hectare was recorded in Quara and Kafta Humera districts respectively (Table 1). These figures are higher than the density of baobab in South Africa (0.8 ± 0.3 stems per hectare) reported by Ref. [37] but lower than the average density in Namibia (3.96–4.13 trees/hectare) reported by Ref. [38]. The highest mean stand density of baobab was also reported by Ref. [30] in different sites in Ethiopia (3.2 ± 0.3 trees per hectare).

**Table 1:** Density and frequency of baobab along three study districts.

Location	Average density of baobab (indiv/ha^−1^)	Frequency of occurrence (%)
Kafta Humera	1.30 ± 0.23	100
Tselemt	3.15 ± 0.15	100
Quara	1.43 ± 0.43	23.33

**Fig. 2:**
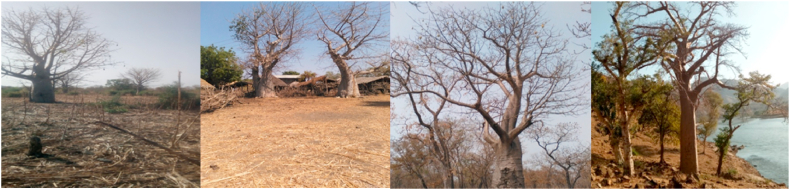
Partial view of sampled baobab trees: Source [45].

In this study, a higher number of baobab tree populations were distributed along the buffer zone of riverine areas, farmland, and homestead land use types (Fig. 2). In contrast, baobab populations were sparsely distributed in forestland use types. Besides, the population of baobab in abandoned villages and church areas was distributed in a clustered pattern. Occurrence of baobab in patches of different densities was reported elsewhere in Taita Taveta County in Kenya [39], Mali [40], and Malawi [41]. In the Tselemt district, the baobab population was clustered along the buffer zone of the Tekeze River, which is an inaccessible and high moisture area (Fig. 2 on the right side). In Quara district, more baobab populations were found close to the human settlements of the farmland and homestead land use types (Fig. 2 on the left side). These might be related to the protection of baobab tree species from browsers including wild animals. Despite this, Assogbadjo et al. [42], Chirwa et al. [43] and Dhillion and Gustad [40] have found that low baobab densities in human‐dominated areas were attributed to livestock browsing and trampling, clearing new fields, fire, and overharvesting of fruit and leaves.

The occurrences of baobab tree species were uniformly distributed in all established sampled plots of Kafta Humera and Tselemt districts (100 %). However, in the Quara district abundant baobab trees occurred only in some established sampled plots (23.33 %) (Table 1).

Baobab tree species were found associated with *Balanytus aegyptiaca, Terminalia laxiflora, Ziziphus mucronata, Acacia senegal, Acacia polyantha, Dalbergia melanoxylon, Combretum adenogonium, Tamarindus indica, Terminalia brownie, Acacia brevispica, Combretum molle* and *Acacia melliferous* in the study areas. *Tamarindus indica* L. is often associated with baobab tree species [44]. Nevertheless, the abundance of tree species associated with baobab is varied from place to place. The associated plant species with baobab in the Omusati region, Namibia are *Hyphaene petersiana, Ficus carica* L. and *Sclerocaraya birrea* L. [38]. Besides, Lisao et al. [12] reported that in Northern Namibia; *Acacia arenarria, Dichrostachys cinerea, Sclerocarya birrea, Ximenia americana, Striculia africana, Terminalia prunioides,* and *Combretum imberbe* species are associated tree species of baobab. In southern Africa, baobab is associated with Colophospermum (mopane), Cordyla, and Kigelia woodlands in low-lying, dry, hot, and frost-free areas [5].

### 3.2 Population structure of baobab stands

**Fig. 3:**
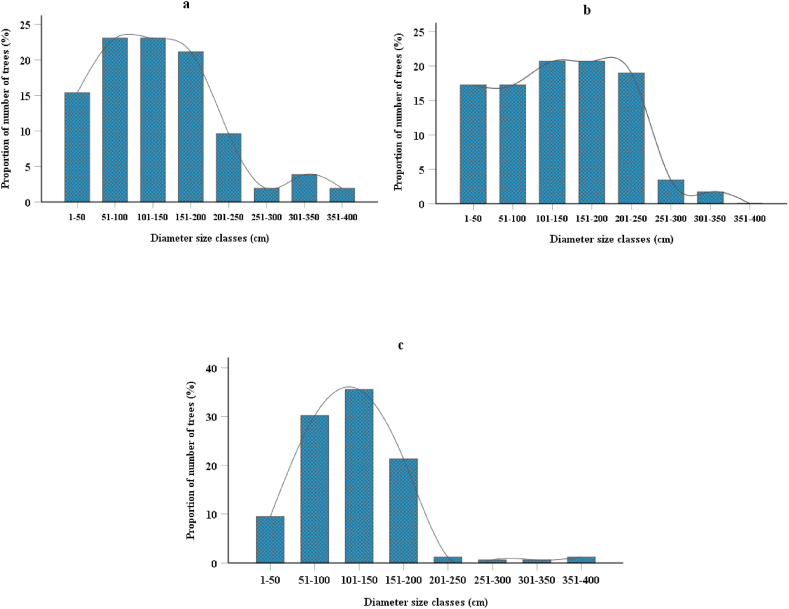
Proportion of baobab trees along the different diameter size class categories of Kafta Humera district (a), Tselemt district (b), Quara district (c).

The population structures of baobab in three-study districts are showed in Fig. 3. The population structure in all three-study areas showed that dominated by matured individuals (Fig. 3). The proportion of diameter size classes, density, and regeneration status are commonly used as indicators of the impact of forest stands [46,47]. All individuals of baobab tree species in all three-study areas have a diameter greater than (3.9 cm). Higher number of young stems were found in Tselemt district (riverine forest stand) compared with the other two study districts (Fig. 3b). These figures supported by Birhane et al. [30] riverine areas had the highest total density of trees (mainly juvenile trees) compared with other land use types. In contrast, the biggest trees of 351–400 diameter size classes were recorded in Quara and Kafta Humera districts (Fig. 3a and c). Higher proportions of baobab were found within the 101–150 cm diameter size class in Kafta Humera and Quara study sites, whereas in Tselemt within the 101–200 cm diameter size class. The population structure in the Quara district showed that bell-shaped distribution (Fig. 3c), which indicates that the population is unstable and under threat due to a lack of regeneration. A similar study in Nambia by Venter and Witkowski [15] presented that low recruitments and bell-shaped distribution are typical of baobab populations. Similar population structures of baobab were reported from across different parts of Africa [12,37,38]. This is due to different anthropogenic factors such as livestock browsing, shifting cultivation practices, and uncontrolled fire [22]. Msalilwa et al. [48] indicated that unsustainable land use type conversions and climate changes could play major roles in reducing baobab distributions. In Tselemt and Kafta Humera, the population structures of baobab revealed that irregularly shaped distribution (Fig. 3a and b) have ununiformed population structure trends. Large ﬂuctuations of population structure along diameter size classes are indicative of an uneven distribution mentioned by Ref. [30].

### 3.3 Regeneration status of baobab

Seedlings and saplings of baobab tree species were lacking in the Quara district sampled plots. Livestock browsing, trampling, and shifting cultivation (forest clearing) practices are the major causal factors for the poor sustainability of baobab recruitment. Similarly, Abere et al. [22] reported that the baobab population was affected by herbivores, forest clearing, fire, and removal of seedlings on farmlands in Quara district, Ethiopia. Moreover, anthropogenic factors that affect baobab recruitments in different land use types [12,30,37,38]. On the other hand, Amusa et al. [49] presented that baobab recruitments are commonly damaged by elephants, in Southern Zimbabwe. In the Kafta Humera district, a few numbers of baobab recruitments were found in open shrub land use type and lacking in the homestead. These low recruitments are a typical baobab population structure across Africa [15,50,51]. However, better recruitments were observed in all sampled plots of the Tselemt district (riverine areas). This is supported by the results of [30] who reported best baobab recruitment was found in the riverine area of other sites in Ethiopia. The riverine areas prevailing high soil moisture contributed to breaking down seed dormancy and facilitating germination, and enhancing the growth of seedlings. Similarly, Lisao et al. [12] presented that denser baobab stands were likewise observed in wetter areas compared to drier areas. Furthermore, seedlings of baobab in the riverine forests have found inaccessible specific sites. These contributed to reducing the browsing and trampling of seedlings by livestock. The regeneration results can be taken as an indicator that baobab has the ability to produce seedlings naturally but these seedlings mostly face difficulties to grow into mature trees. Land clearing and man-made fire are the common practices in farmland uses whereas browsing and trambling are common practices in forests that affect baobab seedlings and saplings in the study areas.

### 3.4 Phenological characteristics of baobab

Baobab has shown six phenophases for its leaf, flower, and fruit periodicity. These phenophases of the baobab tree are presented in Table 2. A similar previous study Dejene et al. [52] displayed that individual deciduous trees exhibited those six phenophase sequences. The pattern of leafing and leaf shade, flowering, and fruiting are unimodal in line with the nature of the rainfall. When the rain is begin in the study areas just the leafing of the baobab commenced and was followed by flowering and fruiting.

**Table 2:** Phenophases of baobab.

No.	Sequential Phenophases		
	Leafing	Flowering	Fruiting
1	Emergence of buds	No inflorescence buds	No fruit
2	Leaf buds initiation	Opening buds	Early fruits
3	Leaf flushing	Opening flowers	Green fruits
4	New leaf	Peak flowering	Fully developed fruits
5	Matured leaf	Withered flowers	Peak fruit maturation
6	Leaf shading	Dried and withered of flowers	Fruit dissemination

The longest Phenological phase was observed from fruiting initiation to harvesting lasting 165 days, whereas the shortest phenophase was flowering, less period existed only for 105 days (Table 3). The leafing phenological phase is the second longest lasting 135 days. Baobab buds burst when the rainy season begins. Similarly, Wickens [5] and Baum [1] indicated that baobab bear leaves and flowers in the wet season and leafless in the dry season. Leaf initiation started in the third week of April and extended up to the end of May. The maximum leaf maturation of baobab occurred between the mid of June and the third week of August month (Table 3). Newly emerged young leaves required 105 days to attain maturity. Matured leaves existed in the summer season (June up to August months). Baobab trees had leafing and flowering phases that coincide with each other in the summer period. Leaf withered and shading continued up to the second weeks of September starting from mid of August. Flowering initiation started from mid of August (from the third week of August up to the middle of September) (Table 3). The full bloom commenced from the third week of September till the mid-week of October. Baobab tree species was taken two months of 60 days to reach full bloom (Table 3). Matured flowers started to wither and dried from the first week of October and ended in the last week of October. During the flowering season, trees produce 10–50 flowers per night [53]. Furthermore, Venter and Witkowski [15] indicated that anthesis usually occurs in the evening and night.

**Table 3:**
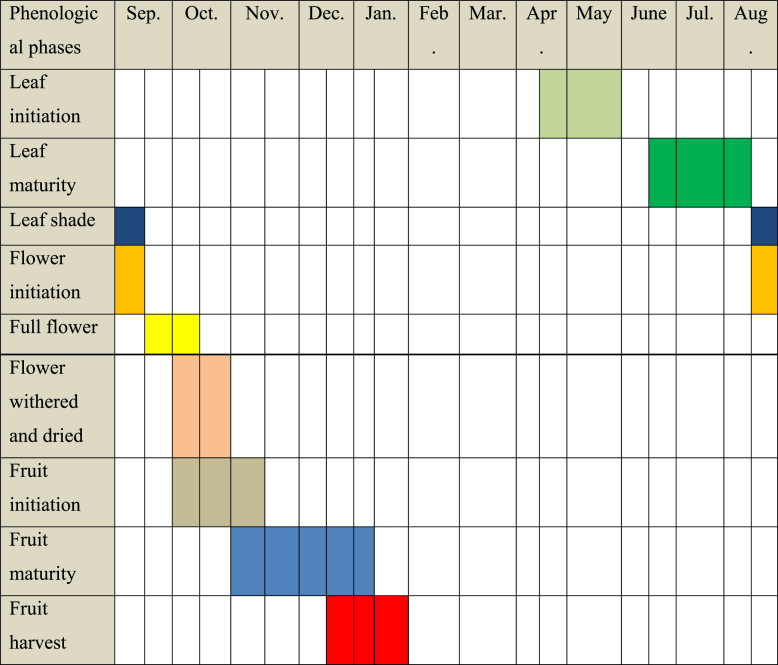
Leaf, flower, and fruit production phenology of baobab in Northwestern lowland of Ethiopia.

Fruiting is the longest Phenological phase, which starts initiation from the first week of October up to mid of November (Table 3). The highest fruit or peak maturation of baobab was observed from the first week of November until mid of January. On average 75 days are needed to complete maturity starting from fruit initiation. These results confirmed the fruit maturation of baobab in December and January [50]. Fruit harvested periods started from the third week of December up to the end of January in northwestern Ethiopia (Table 3). Similarly, Dejene et al. [52] displayed that fruit shading of *S. setigera* in the Metema district, Northwestern Ethiopia was observed to peak in late December and early January. Those results argued that baobab fruits are harvested from May to October in Kenya [54]. In general, to reach fully matured fruits starting from flowering initiation was taken five weeks and 15 days. Sidibe and Williams [2] also reported that the period between flowering and fruit ripening is 5–6 months. Similarly, Sanchez [41] stated that baobab fruits develop 5–6 months after flowering. According to the observation, the bonding that attached baobab fruits with branches is very strong and cannot easily fail without human support. The Phenological period of baobab in three study districts (Kafta Humera, Tselemt, and Quara) was not varied. For example, a flowering of baobab was starting in mid-August and ended in the last week of October in all three study districts (Table 3). Despite this, Sidibe and Williams [2] reviewed the flowering period of baobab in Southern Africa from October–December; November–December in Madagascar, and May–June in Western Africa. Besides, Goldwin [55] and Fenner and Thompson [56] displayed that environmental and biological conditions can have a major impact on the success of fruit sets.

### 3.5 Fruit yield potential of baobab

**Fig. 4:**
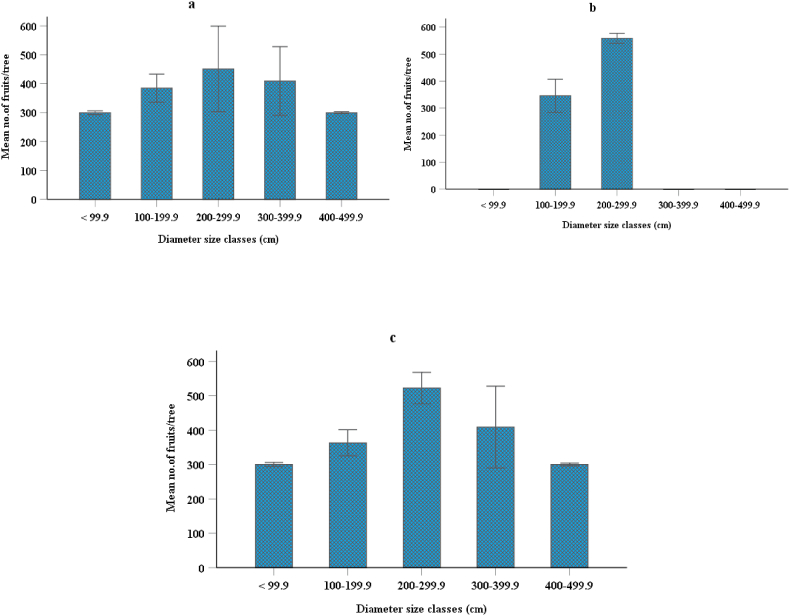
Fruit yield potential of baobab along diameter size class categories of Kafta Humera (a), Tselemt (b), and Quara district (c).

The number of fruits produced per tree did not significantly vary between study districts (P = 0.75). In contrast, Peters [57] and Assogbadjo et al. [50] mentioned that site conditions significantly influenced the baobab fruit productivity. On average, 404 fruit per tree were produced. Each fruit or pod weighed 0.125 kg, so on average, and a tree contained 50.5 kg of fruit. This figure is higher than the average of 250 fruit per tree [58]. Besides, Assogbadjo et al. [50] reported that mean fruit production in Benin varied between 57.1 and 157.4 fruit per tree in different climatic zones. Fruit yields in each area depend on the number of branches. On average, there are seven numbers of branches in each tree and each branch holds 55.3 fruits. The fruit yield potential of baobab along a five-diameter size class was found in all three districts (Fig. 4). Fruit production between the two study years has not much varied in all three study districts. The current study supported by Ref. [37] that fruit produced in each year did not differ significantly. As shown, in the graph higher numbers of fruits were found in the mid-diameter size classes along all three study districts (Fig. 4a, b & c). Baobab trees found in the diameter size class of 200–299.9 cm had better fruit yield than other smaller and bigger diameter size classes (Fig. 4). Similarly, Chapman et al. [59], Botelle et al. [60], Shackleton [61] and Killman et al. [62] suggested that stem diameters could reliably be used to distinguish between sub-adult and adult trees. Furthermore, Venter and Witkowski [37] reported that fruit production has high variability in diameter size classes. A few young and old trees produced fruit in Kafta Humera and Quara districts, whereas in the Tselemt district, there are no young and old trees that produced fruits. Some sampled trees did not produce fruit in the two-year fruiting season, especially at higher-diameter size classes or older trees. Swanepoel [63] reported that over a four-year period baobab in the Mana Pools area of the Zambezi River Valley did not produce any mature fruit. Youngness baobab trees have taken long years to produce fruit.

## 4 Conclusion and recommendations

The size class distribution of matured baobab trees was bell-shaped in the Quara district and irregularly shaped in Kafta Humera and Tselemt reflecting an unhealthy proportion in baobab, which is not stable in districts region mainly due to different anthropogenic factors in Northwestern Ethiopia. Our results indicate a lack of recruitment in the Quara district along with a few numbers in Kafta Humera under shrublands. Better recruitment of the species was noticed in the Tselemt district along the Tekeze River (an inaccessible area from browsers and grazers). The Phenological variations of baobab were not observed between three districts in two consecutive years. The shortest pheno-phase was noticed for flowering that existed only for 105 days from the third week of August–October, whereas, the longest pheno-phase was observed from fruit initiation to harvesting that took 165 days from October–January. The leafing pheno-phase was in between that took 135 days from the third weeks of April to the second weeks of September. Baobab fruit production is not significantly varied between harvested years and study districts. On average 404 numbers of fruits are produced per tree. Higher numbers of fruits are produced in the middle diameter size classes, which are compared with lower and higher diameter size class trees. Finally, the researchers suggest that an in-situ conservation strategy through mass plantation and proper management practices are needed to sustain the distribution of baobab tree species in the study areas.

## Funding statement

This research did not receive any specific grant from funding agencies in the public, commercial, or not-for-profit sectors.

## Data availability statement

Data will be made available on request.

## CRediT authorship contribution statement

**Melkamu Abere:** Writing – review & editing, Writing – original draft, Software, Resources, Project administration, Methodology, Formal analysis, Data curation, Conceptualization. **Zewdu Yilma:** Writing – review & editing, Visualization, Validation, Investigation, Funding acquisition, Data curation. **Tadesse Tsegie:** Writing – review & editing, Visualization, Data curation. **Abeje Eshete:** Writing – review & editing, Software, Project administration, Methodology, Funding acquisition. **Asmamaw Alemu:** Writing – original draft, Methodology, Formal analysis, Conceptualization.

## Declaration of competing interest

The authors declare that they have no known competing financial interests or personal relationships that could have appeared to influence the work reported in this paper.
